# Selecting the Shade

**Published:** 1906-03

**Authors:** Geo. W. Schwartz

**Affiliations:** Chicago.


### SELECTING THE SHADE.

By Geo. W. Schwartz, M. D., D. D. S., Chicago.

No other part of inlay work requires more experience or a greater degree of accuracy than selecting the shade. On it depends the artistic result and its approval by the patient.

In the use of the shade guide all matching should be done before the rubber dam is adjusted. After selecting the shade in natural light compare it also in an artifical one with the room previously darkened if you wish to be critical. View the shade from both sides of the chair at various angles, where the inlay is to be a shallow one the extreme point of the porcelain shade guide should be considered, for this pupose the Brewster company have recently put upon the market an inlay guide with this in mind. In a shallow inlay we will take for instance a labial cavity in the front of the mouth which would perfectly match before setting, would look too light when set, but a trifle brown mixed with the yellow body would have avoided this and this new guide enables you to better judge the colors.

Experience teaches that porcelain fillings for the mesial surfaces of the eight anterior teeth should be a shade darker than the tooth while those of the distal should be a shade lighter.

For second bicuspids and molars it is safe to take a shade darker as a rule with few exceptions. In centrals and laterals for approximal fillings it is also a good rule to follow to use white as a foundation, finishing with the colors desired while nearly all posterior to these should have a yellow foundation, the color of the cusps in almost all bicuspids and molars inclines to blue with few exceptions. In most teeth the cusps are lighter at the point and the body should be laid on with this fact in mind.

The color of the cement should not be overlooked by beginners. It is a suggestion to begin by the use of one color cement and that a yellow, at most not more than two, a yellow and a blue, by these in combination where one or the other is not indicated alone a good result can usually be obtained.

It is not advisable for the beginner to depend much on his ability to select the color of his cement to help the shade of his inlay, if he does he will meet with many disappointments. It is always best to mix a little of the cement powder with water and lay it and the inlay in the cavity, this will give you some idea of how it will look when set, it is discouraging to cement one in and have to remove it, the powder mixed as described obviates this embarrassment. For instance a thick inlay or filling for a yellow tooth that matches it almost perfectly before it is cemented, will after being cemented in place with a yellow cement that matches make it appear to be one or two shades darker than before it was set, the same is true of the blue shade. For this reason it is my belief the beginner can better learn to shade his porcelain to the cement than to depend on his selection of the cement to help an off-colored inlay.

In working colors for cusp restorations for molars and bicuspids, the use of dark brown and dark yellow in the fissure as the case may determine results in good effects, satisfactory and artistic results may be obtained by the use of oil colors for the fissures and buccal margins and without fear are recommended even to the novice, while on the subject of oil colors will for those who have not tried it say it has been my pleasure as well as that of others to learn that Brewster oil colors can be used with water to better advantage than by the use of turpentine.

As a safe method I would advise taking a portion from each bottle of inlay material, glue it to a white card, and fasten it to the bottle, while shades run reasonably true it is desirable to know the *exact* shade of each bottle.

